# Social network structure modulates neural activities underlying group norm processing: evidence from event-related potentials

**DOI:** 10.3389/fnhum.2024.1479899

**Published:** 2024-11-13

**Authors:** Mengfei Han, Ruoxuan Han, Xin Liu, Duo Xie, Rong Lin, Yaokun Hao, Hanxiao Ge, Yiwen Hu, Yuyang Zhu, Liu Yang

**Affiliations:** ^1^Aviation Psychology Research Office, Air Force Medical Center, Fourth Military Medical University, Beijing, China; ^2^Research Institute of Law, Sichuan Academy of Social Sciences, Chengdu, China

**Keywords:** social network, group norm, social feedback, brain network, ERP

## Abstract

**Introduction:**

Social ties play a crucial role in determining the health and wellbeing of individuals. However, it remains unclear whether the capacity to process social information distinguishes well-connected individuals from their less-connected peers. This study explored how an individual’s social network structure influences the dynamic processing of group norms, utilizing event-related potentials (ERPs).

**Methods:**

The study involved 43 university students from the same class who participated in a social network study measuring metrics such as real-life social network size, in-degree, out-degree, and betweenness centrality. Subsequently, 27 students participated in an EEG study assessing their willingness to engage in various exercises after being exposed to peer feedback or in its absence.

**Results:**

The results indicate that an individual’s social network structure is significantly associated with the dynamic processing of group norms. Notably, well-connected individuals exhibited larger ERP amplitudes linked to feedback (e.g., N200, P300, and LPP), greater functional segregation within the brain network (e.g., local efficiency and clustering coefficient), and enhanced synchronization within frontal area and across different brain areas.

**Discussion:**

These findings highlight that well-connected individuals possess enhanced sensitivity and efficiency in processing social information, pointing to potential areas for further research on the factors influencing social network evolution.

## 1 Introduction

The dynamic and diverse social connections in daily life form our social network. The social network structure of an individual, such as network size and complexity, provides insights into how an individual navigates within their social world and reflects their role within a social group (Baek et al., 2022; Han et al., 2021). Moreover, it is widely recognized as a critical determinant influencing individuals’ health and wellbeing (Bryant et al., 2017; Mori and Haruno, 2021).

Can the potential impacts of social network structures on individuals extend to aspects of social cognitive processing? Previous studies found that individuals occupying various core-periphery positions within a social network demonstrate differing cognitive processes or capacities (Brashears et al., 2020). Well-connected Individuals, such as those with numerous friends or with many opportunities to connect with otherwise isolated individuals, are typically central-positioned in their social network and have enhanced opportunities for information exchange and complex social scenarios exposure (Han et al., 2021; Han et al., 2023). In order to establish and maintain their prominent role within the social group, well-connected individuals must process social information, such as understanding the thoughts, perspectives, and mental states of others, more rapidly and efficiently (Stiller and Dunbar, 2007; O’Donnell et al., 2017). Nonetheless, it is less clear how the social cognitive processes of individuals vary based on their social network structure within a specific group. In-depth explorations of social networks and their potential impacts on social cognitive processes may yield a more profound comprehension of the distinguishing factors that can set well-connected individuals apart from other individuals.

Numerous studies have utilized various social tasks to investigate how social networks modulate brain activity during social cognition processes. Previous research has shown that larger and more complex personal social network structures enhance task performance, including increased sensitivity to biological motion (Kirby et al., 2018), improved accuracy in perspective-taking (Meyer and Lieberman, 2016), increased consideration of other people’s opinions in social influence task (O’Donnell et al., 2017), enhanced social working memory (Krol et al., 2018) and better response inhibition in go/no-go tasks (Tompson et al., 2020). Furthermore, social network structure has been identified as a positive predictor of neural activity and functional connective strength in brain regions associated with social cognition, such as the medial prefrontal cortex, orbitofrontal cortex, and temporoparietal junction, during the execution of these social tasks (Baek et al., 2022; Krol et al., 2018; Meyer and Collier, 2020; O’Donnell et al., 2017; Stiller and Dunbar, 2007). However, despite these findings, previous studies have yet to deconstruct the social cognition processes. Therefore, the current study employed electroencephalography (EEG) with high temporal resolution to gain a deeper understanding and refine the knowledge of how the dynamic process underlying social cognition varies with social network structure.

This study focused on processing group norms as a critical social cognitive process. The recognition and adherence to group norms are essential for the successful establishment and maintenance of social relationships, suggesting that well-connected individuals may exhibit greater sensitivity to their peers’ norms, either as a causal factor or as a consequence of their network centrality (Baek et al., 2020; Han et al., 2023). The processing of group norms by individuals may involve the detection of the gap between self and others (i.e., self-group conflict), the evaluation of whether to approach or avoid the group based on these conflicts, and the final decision regarding whether to adjust behaviors to align with group norms (Shamay-Tsoory et al., 2019). In prior research, group norms are usually presented as feedback. Based on these studies, it has been observed that components in event-related potentials (ERPs) like N200, P300, and LPP exhibit differences when individuals encounter group norms as social feedback in contrast to conditions where group norms are absent (Chen et al., 2012; Shestakova et al., 2013; Wang et al., 2019). The N200 component is a negative-going wave and peaks around 250–300 ms following the stimulus onset. The frontocentral N200 is involved in the anticipation of violation, negative feedback, and cognitive conflict, while posterior N200 is associated with visual attention to perceptual mismatch (Folstein and Petten, 2008; Wang Y. et al., 2020; Xie et al., 2016). The P300 wave, a positive-going wave occurring approximately 300–500 ms post-stimulus onset, has been linked to processing feedback information for decision-making processes. Moreover, the P300 amplitude is commonly positively correlated with subsequent behavioral alterations (Valt et al., 2020; Han et al., 2023). The late positive potential (LPP), a continuous positive deflection beginning around 300 ms after stimulus presentation, reflects sustained attention processes and later phases of emotional evaluation during the reevaluation of social feedback. Notably, heightened LPP amplitudes are associated with social reward, which may be induced by conforming to group norms (Li et al., 2022; Schindler et al., 2015).

Previous studies have identified the dissociative effects of social network structure on EEG components evoked by social feedback. In one of our recent studies, we manipulated individuals’ social network structures by assigning them to exercise support groups comprising either their nominated friends (high network centrality) or non-nominated classmates (low network centrality). Our findings demonstrated that in comparison to conditions without group norm presentation, self-group conflicts triggered a heightened negative-going feedback-related negativity wave, observed solely in the high network centrality group rather than the low network centrality group. Interestingly, the effects of self-group conflicts on P300 did not exhibit discrepancies between the two groups (Han et al., 2023). These findings suggest that an individual’s position within the social network influences their susceptibility to group norms without inducing subsequent behavioral alterations. However, it is worth noting that this study artificially manipulated individuals’ social network structures without utilizing real-life social network metrics.

In the present study, beyond focusing on ERP components, we also investigated the connectivity efficiency of task-related functional brain networks constructed based on EEG synchronization across different frequency bands (Wang Y. et al., 2020). Brain networks exhibit complex structures and topological characteristics that can comprehensively depict cognition-related information exchange patterns and efficiency (Li et al., 2020). Numerous studies have confirmed that EEG frequency bands are crucial neural indicators for various social processes such as social cognition, communication interactions, social function, emotional engagement, etc. Notably, the low-frequency bands activities, including alpha, delta, beta, and theta, are susceptible to negative social feedback (e.g., threats, conflicts) and are associated with subsequent reward evaluation and behavior adjustment (Balconi et al., 2018; Cavanagh, 2015; Leitner et al., 2014). On the other hand, high-frequency bands like gamma are implicated in processing feed-forward prediction errors and conceptual/behavioral synchronization (Chen et al., 2022; van Pelt et al., 2016). However, minor muscle artifacts often contain power predominantly in the gamma band. Consequently, many ERP studies prefer to apply a low-pass filter to EEG signals at 30 Hz to mitigate these artifacts (Balconi et al., 2018; Fries et al., 2008). Due to concerns regarding motion artifacts, our study excludes the gamma band from analysis. Therefore, our study integrated ERP components and brain networks to delve deeper into the impact of an individual’s social network structure on social cognitive processing.

The present study aims to elucidate the associations between social network structure and dynamic neural processing underlying social cognition in exercise contexts. Previous studies have found that group norm is one of the most important determinants of individual exercise behavior (Chung and Rimal, 2016). Observing peers in social networks engaging in leisure-time physical activities can enhance one’s motivation and participation in exercise (Ball et al., 2010; Hamari and Koivisto, 2015; Okun et al., 2003). Understanding how social norms influence individual exercise behaviors is essential for creating effective intervention strategies that can promote exercise. This insight holds potential value for practical application. To achieve this goal, we initially substituting the traditional consumption scenario in the classic social influence experiment paradigm with an exercise scenario (O’Donnell et al., 2017). Then, we employed multiple social network metrics to measure the extent of social connectedness, including an individual’s real-life social network size (i.e., the number of friends who can provide social support) as well as their social network structure within a specific group (e.g., in-degree, out-degree, and betweenness; see details below). Moreover, our study incorporated event-related potentials (ERPs) and EEG-based functional brain networks to comprehensively explore individuals’ dynamic cognitive processing during a social influence task. We hypothesized that individuals with stronger connections would exhibit heightened sensitivity to self-group conflicts on exercise willingness and demonstrate increased efficiency in brain functional connectivity when making decisions during conflict situations.

## 2 Methods

### 2.1 Participants

A total of 43 university students, comprising 30 females and 13 males with a mean age of 20.81 years (*SD =* 0.54), completed the initial social network questionnaire. All 43 subjects were from the same class, which had a total of 44 members, resulting in a response rate of 97.72%. Among them, 27 students voluntarily participated in the subsequent behavioral and ERP experiment (six males; mean age = 20.85 years, *SD =* 0.53 years), while the remaining did not participate in any follow-up experiments. They were all right-handed, had normal or corrected vision, and had no history of psychiatric illness. All participants provided informed consent in accordance with the Institutional Review Board of Beijing Sport University guidelines and were treated in accordance with the Declaration of Helsinki.

### 2.2 Materials

*Stimuli:* A comprehensive set of 60 color images depicting distinct exercise behaviors were used as the experimental stimuli. The exercise behaviors included outdoor and indoor sports, such as swimming, strength training, etc. None of the pictures depicted explicit affective content, such as smiling faces (Han et al., 2023; Schinkoeth et al., 2019). The development of the experimental stimuli and their characteristics are shown in the Supplementary material.

*Social network metrics*: Social network metrics were assessed as follows:

1. Real-life social network size: The size of an individual’s real-life social network was measured by the Social Network Questionnaire developed by Lewis et al. (2011). Participants were requested to list their friends and family members with whom they maintain frequent contact (at least once every 2 weeks). The real-life social network size was defined as the total number of listed individuals;
2. In-class social network indicators: Participants were requested to nominate their particularly close friends within their class (Han et al., 2023; Hyon et al., 2020). Participants were asked to nominate at least one and no more than five friends to ensure that these nominations accurately reflected “particularly close” friendships. Based on the nominations provided by the 43 participants, an in-class social network was constructed. Subsequently, we analyzed three common social network indicators based on the in-class social network. For instance, degree centrality refers to the number of direct connections to an individual, including in-degree (number of received connections) and out-degree (number of sent connections). Meanwhile, betweenness centrality refers to the frequency with which an individual occupies the shortest paths connecting pairs of other individuals within the network (Han et al., 2023).

### 2.3 Procedure

**Figure 1:**
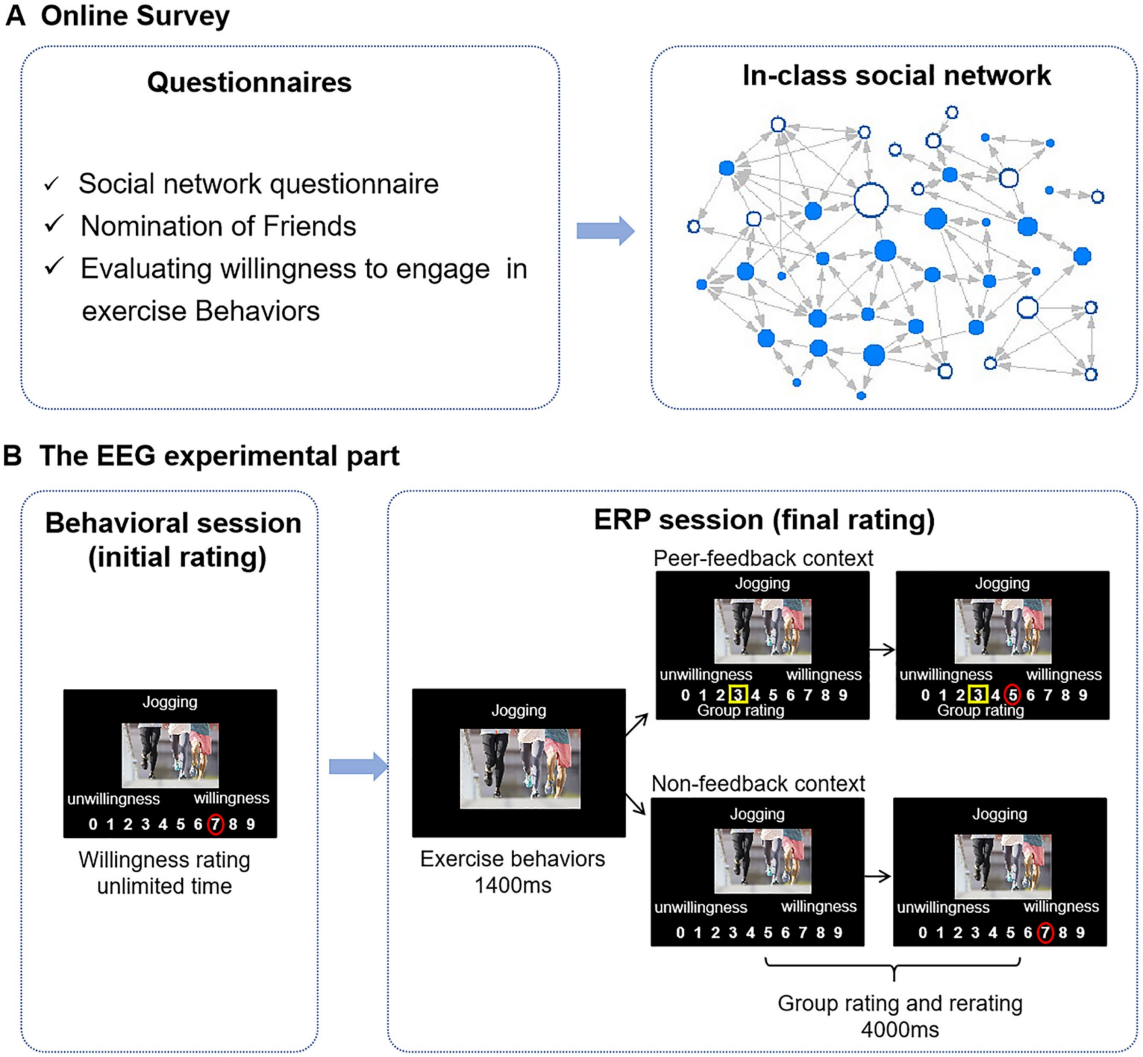
Flowchart of the experiment. (A) A week before the EEG experiment, participants were required to fill out a series of questionnaires online. The in-class social network is constructed based on “nomination of friends.” Each node represents an individual student, whereas each connecting line represents a reported social tie. Directed edges are illustrated through arrows, where an arrow pointing from node A to node B signifies that student A has nominated student B as a friend. The size of a node represents students’ betweenness centrality, with larger nodes indicating higher betweenness centralities. Blue nodes denote ERP study participants. (B) In the behavioral session, participants were asked to rate their willingness to engage in the exercise behaviors shown on the stimulus images. In the ERP session, participants were asked again to rate the same stimulus images, presented with group ratings that differed from participants’ initial ratings (peer-feedback context) or without (non-feedback context). Group ratings are highlighted with yellow rectangles, and individuals’ ratings are presented with red circles.

*Online survey:* One week before the ERP experiment, participants enrolled in the same class were tasked with completing the Social Network Questionnaire, the nomination of friends, and the IPAQ-SF. In addition, these participants were also required to evaluate their willingness to engage in the exercise behaviors depicted in 20 randomly chosen images from the 60-experiment stimulus set described above. This assessment was conducted using a 10-point scale ranging from 0, indicating very unwilling, to 9, indicating very willing (see Figure 1). The purpose of this evaluation was to enhance the credibility of the experiment. In the subsequent ERP experiment, the participants were informed that group ratings were based on this survey.

*Behavioral session (initial rating)*: Participants voluntarily participated in the subsequent ERP experiment and arrived at the laboratory 1 week later. Before the formal ERP experiment, participants were asked to rate their willingness to engage in the exercise behaviors presented on the 60-experiment stimulus on a 10-point scale (ranging from 0 to 9) by clicking the corresponding number with a mouse (initial rating). The experimental procedure was administered via E-prime 2.0 software, with the stimulus images centrally displayed on the screen against a black background and the rating scales positioned below. The stimuli were presented in a random order. There was no time limit for the participants to make a decision (see Figure 1).

*ERP session (final rating)*: Subsequently, participants proceeded to engage in the ERP experiment. In the ERP experiment, participants were asked to evaluate their willingness to exercise behaviors based on the same 60-experiment stimulus as that in the initial rating (final rating) again. The 60 trials were divided into two conditions: 30 trials in the peer-feedback context and 30 trials in the non-feedback context.

In the peer-feedback trials, participants viewed how their classmates rated the 60-experiment stimulus (group rating) before they rated. Participants were informed that the group ratings were the average rating of their classmates in the online survey. In reality, group ratings were experimentally manipulated based on the participants’ initial ratings during the behavioral session to induce social influence. Using an adaptive algorithm, the group rating was randomly adjusted to be 1, 2, or 3 points above or below the participant’s initial rating. For instance, if a participant’s initial rating was 2, and the group rating was set to 3 points higher in the predesigned procedure, the presented group rating would be 5. We tried evenly distributing the number of trials across each condition (± 1, ± 2, ± 3). Nonetheless, there may still be some extreme trials. The updating direction was reversed if the group rating exceeded nine or dropped below 0 in a trial. For instance, if a participant’s initial rating was 2, and the group rating would display a difference of 3 points lower in the predesigned procedure, the group rating presented to the participant would be 5 (i.e., 2 + 3). Each trial was initiated with a fixation cross on the screen for 800–1,000 ms, followed by the display of stimulus images for 1,400 ms. Then, a 10-point scale ranging from 0 to 9 was shown under the stimulus picture with a yellow rectangle indicating the group rating for 4,000 ms. Participants were asked to decide within this 4,000 ms. In the non-feedback trials, group ratings were not presented (Huang et al., 2014; see Figure 1). All 60 trials were presented in a random order and intermixed.

### 2.4 EEG data recording and analysis

The EEG data were acquired by 64-channels Ag/AgCl electrodes based on the international 10–20 system (NeuroScan Inc., USA) and were recorded online by the Curry 7.0 software platform. The left and right mastoids served as reference electrodes, with the forehead as grounded. To avoid the interference of eye links and movements, electrodes were placed on the lateral side of both eyes to capture horizontal electrooculogram (HEOG) and above and below the left eye to capture vertical electrooculogram (VEOG). The impedance between the scalp electrodes was consistently maintained below 5kΩ during data acquisition. All electrode recordings were amplified using a filter band pass of 0.05–100 Hz and a sampling rate of 500 Hz.

Subsequently, the EEG data underwent offline analysis using EEGLAB 19.0. The data were filtered with a high-pass filter set at 0.1 Hz and a low-pass filter at 30 Hz. The whole-brain average was served as a reference. Ocular artifacts were automatically corrected through independent component analysis (ICA) to ensure the data’s accuracy and reliability.

#### 2.4.1 ERP data processing

The ERP analysis epoch was from 200 ms before the onset of each stimulus, serving as the baseline, and ended 1,000 ms after the image presentation. The image was presented either with group ratings below it in the feedback context or without group ratings in the non-feedback context (see Figure 1). Artifact removal using a threshold of ±75 μV.

**Figure 2:**
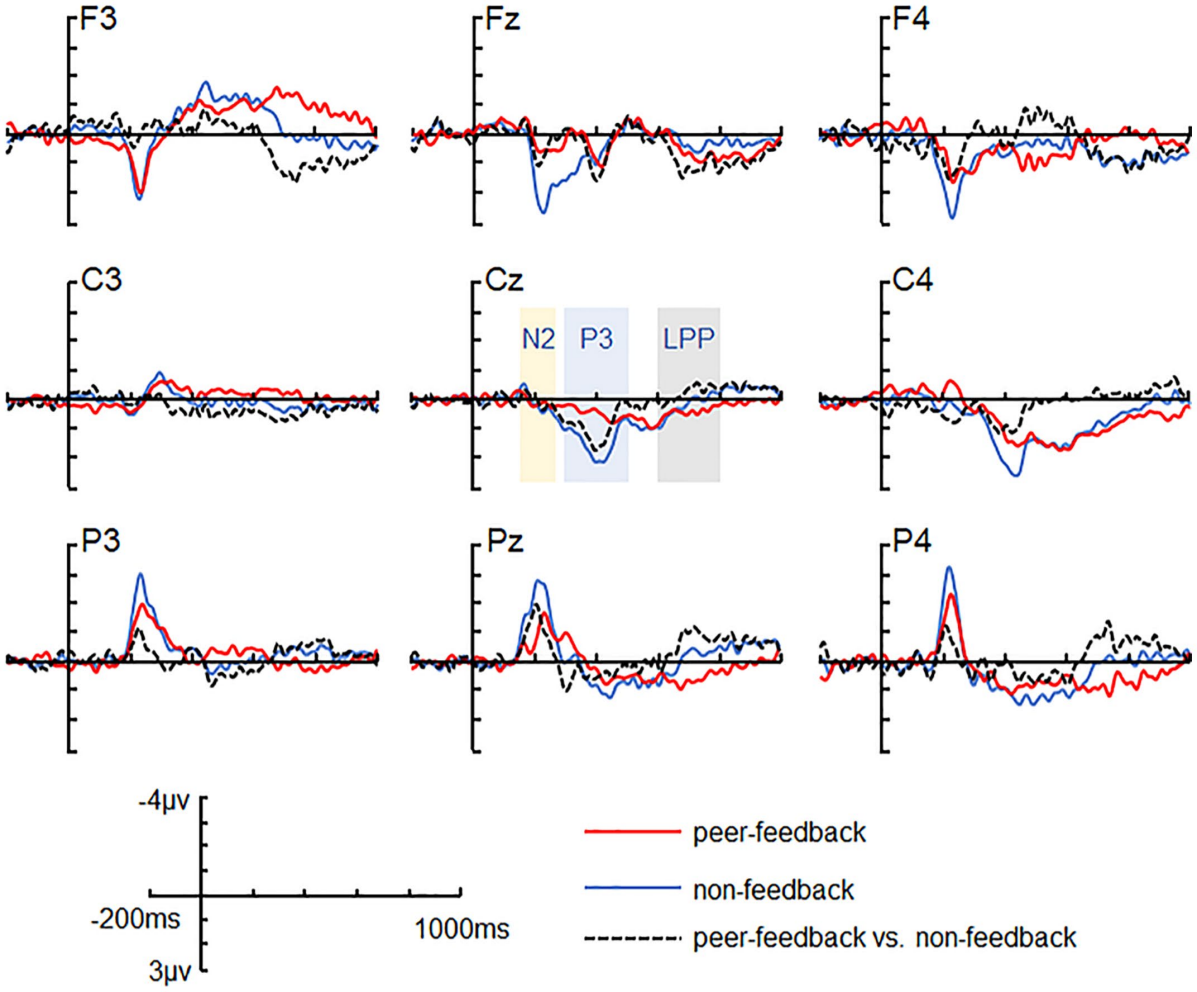
ERP grand-average waveforms. Grand-average waveforms at channels F3, FZ, F4, C3, CZ, C4, P3, PZ, and P4 for peer-feedback, non-feedback and difference waves (peer-feedback minus non-feedback).

**Figure 3:**
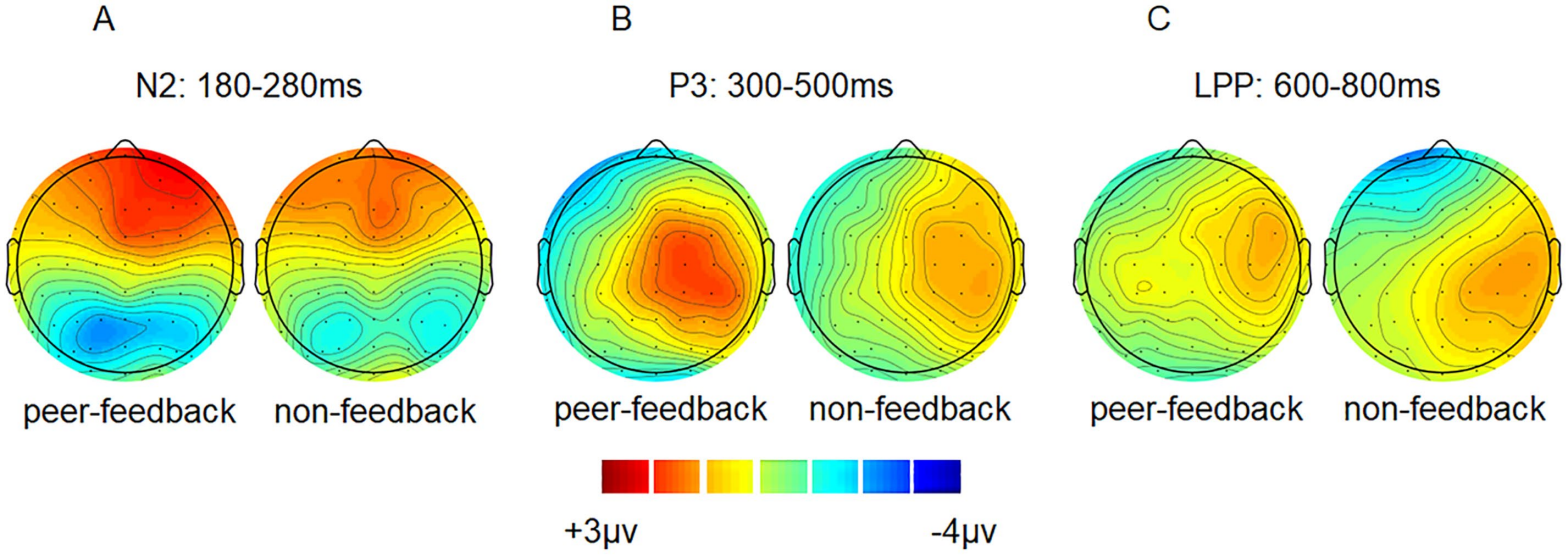
ERP topographical maps. Topographical maps of peer-feedback, non-feedback and difference waves (peer-feedback minus non-feedback) at 180–280 ms (A), 300–500 ms (B) and 600–800 ms (C).

Based on previous studies and visual inspection (Chen et al., 2012; Xie et al., 2016), we analyzed FRN, P3, and LPP amplitude within specific time windows (180–280 ms, 300–500 ms, and 600–800 ms) by averaging the ERPs’ amplitudes across all trials in both peer-feedback and non-feedback contexts for each participant, respectively. Additionally, FRN_diff_, P3_diff_, and LPP_diff_ were calculated as the difference in average amplitudes between two conditions (peer-feedback minus non-feedback). Nine brain regions were considered: left frontal (F3, F5, F7, FC3, FC5, FT7), left central (C3, C5, CP3, CP5, TP7), left parietal (P3, P5, P7, PO5, PO7, O1), medial frontal (F1, FZ, F2, FC1, FCZ, FC2), medial central (C1, CZ, C2, CP1, CPZ, CP2), medial parietal (P1, PZ, P2, PO3, POZ, PO4), right frontal (F4, F6, F8, FC4, FC6, FT8), right central (C4, C6, CP4, CP6, TP8) and right parietal (P4, P6, P8, PO6, PO8, O2) area (Han et al., 2023) (Figures 2, 3).

We conducted a three-way repeated-measures ANOVA with factors of social feedback (peer-feedback and non-feedback), hemisphere (left, medial, and right), and region (frontal, central, and parietal) to analyze the FRN, P3, and LPP amplitudes. Greenhouse-Geiser correction was applied to adjust all *p*-values. Subsequently, Pearson correlation analysis was performed to explore the associations between FRN_diff_, P3_diff_, and LPP_diff_ with individual social network metrics, including real-life social network size, in-degree, out-degree, and betweenness centrality. The multiple correlations were adjusted with the Benjamin-Hochberg False Discovery Rate (FDR) method, a widely preferred and efficient method for FDR control (Benjamini and Hochberg, 1995).

Significant differences in ERP responses between the peer-feedback and non-feedback contexts were further identified using a non-parametric cluster-based permutation test, as implemented in the FieldTrip toolbox (Maris and Oostenveld, 2007). We performed paired sample t-tests to compare the ERP responses elicited by the two conditions at each electrode and sampling point. T-values were considered significant at a level of 0.05, and significant *t*-values were grouped into clusters based on spatial (electrodes) and temporal (sampling points) adjacency. The t-values within each cluster were then aggregated, and Monte Carlo p-values were computed based on 5,000 random partitions for each cluster individually.

#### 2.4.2 Functional brain network analysis

In order to examine the impact of individual social network metrics on brain network efficiency, functional brain networks were constructed using Hermes (Niso et al., 2013) within both peer-feedback and non-feedback contexts across various frequency bands. These bands included delta (0.5–4 Hz), theta (4–8 Hz), alpha (8–12 Hz) and beta (14–20 Hz), utilizing pre-processed EEG signals. The EEG epoch ranged from the onset of each stimulus to 1,000 ms after stimulus presentation. Within the brain network, EEG channels (excluding CB1, CB2, M1, and M2) were defined as nodes, while the connection strength between nodes represented the links. The phase lock value (PLV) was used to quantify the connection strength between node pairs. Specifically, PLV captures the phase synchrony of EEG signals and is calculated as the absolute value of the instantaneous phase difference between two EEG signals (Lei et al., 2012). PLV ranges from 0 to 1, with higher values indicating greater synchronization of EEG signals between the two channels.

To assess the general functional characteristics of brain networks, we utilized graph theory to calculate the topological characteristics of each brain network using the GRETNA toolbox in Matlab.^1^ The topological characteristics were derived from binarized brain networks with sparsity levels ranging from 0.05 to 0.5 in steps of 0.05 (Zhang et al., 2021). We analyzed the area under the curve (AUC) for global efficiency (GE), clustering coefficient (CC), assortativity coefficient (AC), and local efficiency (LE) across two experiment conditions within each of the four frequency bands. The definitions of the topological characteristics are as follows (Wang Z. et al., 2020):

1. GE is the inverse of the average shortest path lengths among all pairs of nodes in the network, indicating the overall efficiency of information exchange throughout the brain network.
2. CC is the average ratio of actual connected edges to all possible connected edges between neighboring nodes, indicating the capacity for specialized processing within local brain regions.
3. AC is the correlation between the degrees (number of direct connections to other nodes) of the connected node pairs, indicating the similarity in connection patterns between different brain regions.
4. LE is the global efficiency of the network consisting of its neighbors after removing a specific node, indicating the efficiency of information transfer within a local brain region.

We used SPSS (27.0 for Windows; SPSS Inc.) for statistical analyses. Firstly, paired *t*-tests were conducted to compare differences in brain network characteristics between the peer-feedback and non-feedback conditions across the four frequency bands. Secondly, Pearson correlation analyses were conducted to evaluate the associations between brain network characteristics and social network metrics.

Additionally, individual functional connectivity strength (i.e., PLV) between all pairs of EEG channels in each brain network was considered. We calculated the differences in PLV between two experimental conditions (ΔPLV: peer-feedback vs. non-feedback) within the four frequency bands. Then, the GRETNA toolbox was used to perform the Pearson correlation analysis between ΔPLV values and social network metrics.

## 3 Results

### 3.1 Behavioral measures

#### 3.1.1 Social network metrics

The Pearson correlation analysis revealed that individuals’ real-life social network size positively correlated with in-class social network indicators (e.g., individuals’ in-degree centrality). Individuals with high betweenness centrality in their class tended to have higher level of popularity (i.e., in-degree centrality) and sociability (i.e., out-degree centrality). Additionally, a positive relationship was observed (see Table 1).

**Table 1:** Correlation analysis among social network metrics.

	Social network metrics	*M* (*SD*)	1	2	3
1	Real life social network size	9.33(3.57)	1		
2	Out-degree centrality	3.07 (1.71)	0.59^**^	1	
3	In-degree centrality	3.11 (1.34)	0.20	0.59^**^	1
4	Betweenness centrality	3.33 (3.09)	0.31	0.67^***^	0.55^**^

#### 3.1.2 Reaction time (RT)

The paired *t*-test results indicated a significant increase in RTs under the peer-feedback context (*M =* 1371.77 ms, *SD =* 363.02 ms) compared to the non-feedback context [*M =* 1204.46 ms, *SD =* 250.45 ms; *t*(26) = 3.66, *p* < 0.001, the effect size of *cohen*’*d* = 1.44]. Pearson correlation analyses examined the relationship between RTs and social network metrics, including real-life social network size, in-degree centrality, out-degree centrality and betweenness centrality. However, no statistically significant effects were observed in the correlation analysis.

#### 3.1.3 Influence score

We defined the influence score as the proportion of trials in which an individual modified his/her choice in the final rating under the peer-feedback context or non-feedback context (Han et al., 2023). The influence score was used to evaluate how much the group norm influenced the participant. Paired *t*-test revealed a significantly increased influence score in the peer-feedback context (*M =* 34.57%, *SD =* 19.88%) than non-feedback context [*M =* 24.32%, *SD =* 22.41%; *t*(26) = 2.58, *p =* 0.016, the effect size of *cohen’d* = 1.01]. Pearson correlation analyses examined the relationship between influence score and social network metrics. However, no statistically significant effects were observed in the correlation analysis.

Furthermore, *conforming trials* were operationally defined as during the ERP session in which participants adjusted their initial ratings to align with the group feedback (Han et al., 2023). The percentage of conforming trials observed within the peer-feedback context, indicative of participants’ tendency to conform, was 72.60% (*SD =* 26.15%). Pearson correlation analyses revealed a significant negative correlation between participants’ tendency to conform and betweenness centrality in the social network (*r =* −0.43, *p =* 0.026). Additional findings regarding the behavior adjustment between initial and final ratings can be found in the Supplementary Material.

### 3.2 ERP results

#### 3.2.1 N2 (180–280 ms)

For the N2 amplitude, the main effect of social influence was significant (see Table 2). Specifically, the peer-feedback context (*M =* −1.78, *SE = 0*.11) evoked a more negative-going wave than the non-feedback context (*M =* −1.47, *SE = 0*.10). Additionally, a significant interaction between social influence and the region was observed. Further post-hoc analysis revealed a main effect of social influence in the parietal area [*F*_(1,62)_ = 14.08, *p* < 0.001, $\eta_{p}^{2}$ *=* 0.17; mean ± SE: peer-feedback, −3.52 ± 0.29 μV; non-feedback, −2.55 ± 0.28 μV], with no significant effects observed in the frontal or central area.

**Table 2:** ERP results of repeated-measures ANOVAs.

	df	N2 (180–280ms)			P3 (300–500ms)			LPP (600–800 ms)		
		*F*	*p*	$\eta_{p}^{2}$	*F*	*p*	$\eta_{p}^{2}$	*F*	*p*	$\eta_{p}^{2}$
S	1,26	**12.59**	**0.001**	**0.32**	**32.13**	**<0.001**	**0.55**	< 1		
S × R	2,52	**7.76**	**0.007**	**0.23**	1.92	0.175	0.07	**6.89**	**0.009**	**0.21**
S × H	2,52	3.05	0.076	0.11	1.69	0.20	0.06	1.42	0.252	0.05
S × R × H	4,104	2.37	0.110	0.08	1.20	0.31	0.04	1.58	0.206	0.06

S, social feedback; R, region; H, hemisphere. Significant effects are marked in bold.

Further correlation analyses were conducted to explore the relationship between N2_diff_ amplitude and social network metrics across all nine brain regions. A negative correlation was observed between real-life social network size and N2_diff_ amplitude in the left central area (*r =* −0.42, *p =* 0.030). In terms of betweenness centrality, a positive relationship was found between betweenness centrality and N2_diff_ amplitude in the right frontal area (*r =* 0.41, *p = 0*.032). Conversely, negative correlations were found between betweenness centrality and N2_diff_ amplitude in the left central area (*r =* −0.45, *p =* 0.019) and left parietal area (*r =* −0.42, *p =* 0.030). However, after FDR correction (*p* < 0.05), no results retained their statistical significance. Increasing the FDR significance threshold to 0.1, however, maintains the statistical significance of main findings. For instance, there is a notable relationship between betweenness centrality and both N2_diff_ amplitude observed in the left central (*p_FDR_* = 0.096) and parietal areas (*p_FDR_* = 0.096). The trends in how brain activities during group norm processing are influenced by the individuals’ social network structure exist. The trends indicating that brain activities during group norm processing are affected by the individuals’ social network metrics.

#### 3.2.2 P3 (300–500 ms)

**Figure 4:**
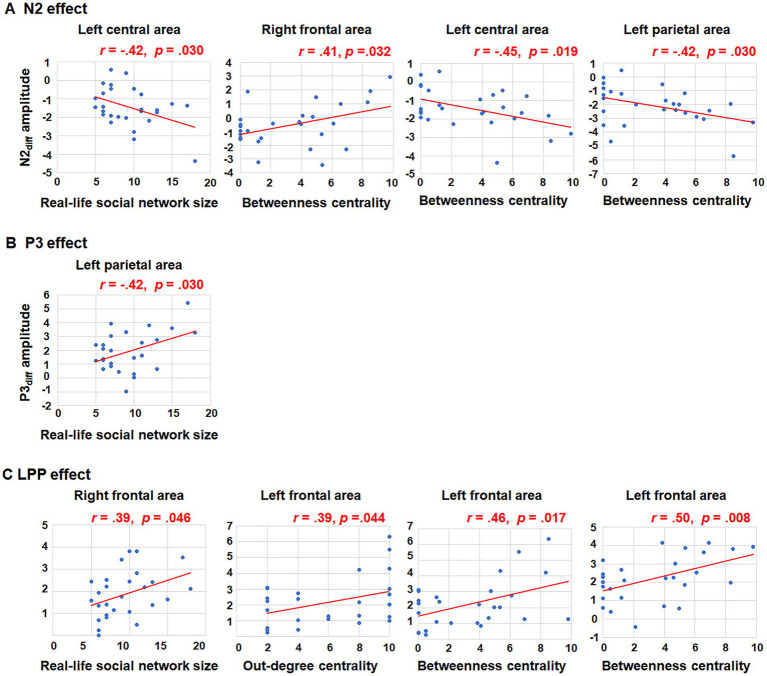
Correlations between social network metrics and N2_diff_, P3_diff_, LPP_diff_ amplitudes. Only significant relationships were presented. **(A)** The significant correlations between N2_diff_ amplitude and social network metrics in the left central area, right frontal area and left parietal area; **(B)** The significant corrections between P3_diff_ amplitude and social network metrics in the left parietal area; **(C)** The significant corrections between LPP_diff_ amplitude and social network metrics in the right and left frontal area.

For the P3 amplitude, RT-ANOVA revealed a significant main effect of social influence, as outlined in Table 2. An increased P3 amplitude was observed in the peer-feedback context compared to the non-feedback context (mean ± *SE*: peer-feedback, 1.84 ± 0.09 μV; non-feedback, 1.54 ± 0.07 μV). No other effect was statistically significant in the analysis. The correlation analysis revealed a positive correlation between P3_diff_ amplitude and real-life social network size in the left parietal area (*r =* 0.42, *p =* 0.030, *p* = 0.270; see Figure 4). No correlations remained significant after FDR correction.

#### 3.2.3 LPP (600–800 ms)

For the LPP amplitude, a significant interaction of social influence and region was observed (see Table 2). Post-hoc analysis indicated a significant main effect of social influence in the frontal area [*F*_(1,62)_ = 13.46, *p* < 0.001, $\eta_{p}^{2}$ *=* 0.34], with peer-feedback context evoked a more positive going LPP wave compared to the non-feedback context (mean ± SE: peer-feedback, 1.46 ± 0.16 μV; non-feedback, 0.94 ± 0.09 μV).

Further correlation analyses were conducted on LPP_diff_ amplitude and social network metrics across all nine brain areas. Significant positive correlations were observed between LPP_diff_ amplitude and real-life social network size in the right frontal area (*r =* 0.39, *p =* 0.046), out-degree centrality in the left frontal area (*r =* 0.39, *p =* 0.044), betweenness centrality in the left frontal area (*r =* 0.46, *p =* 0.017) and betweenness centrality in the medial frontal area (*r =* 0.50, *p =* 0.008; see Figure 4). The *p*-values of correlations between betweenness centrality and both LPP_diff_ amplitude observed in the left (*p_FDR_* = 0.076) and medial (*p_FDR_* = 0.072) frontal areas were <0.1.

#### 3.2.4 Results of the cluster-based permutation test

**Figure 5:**
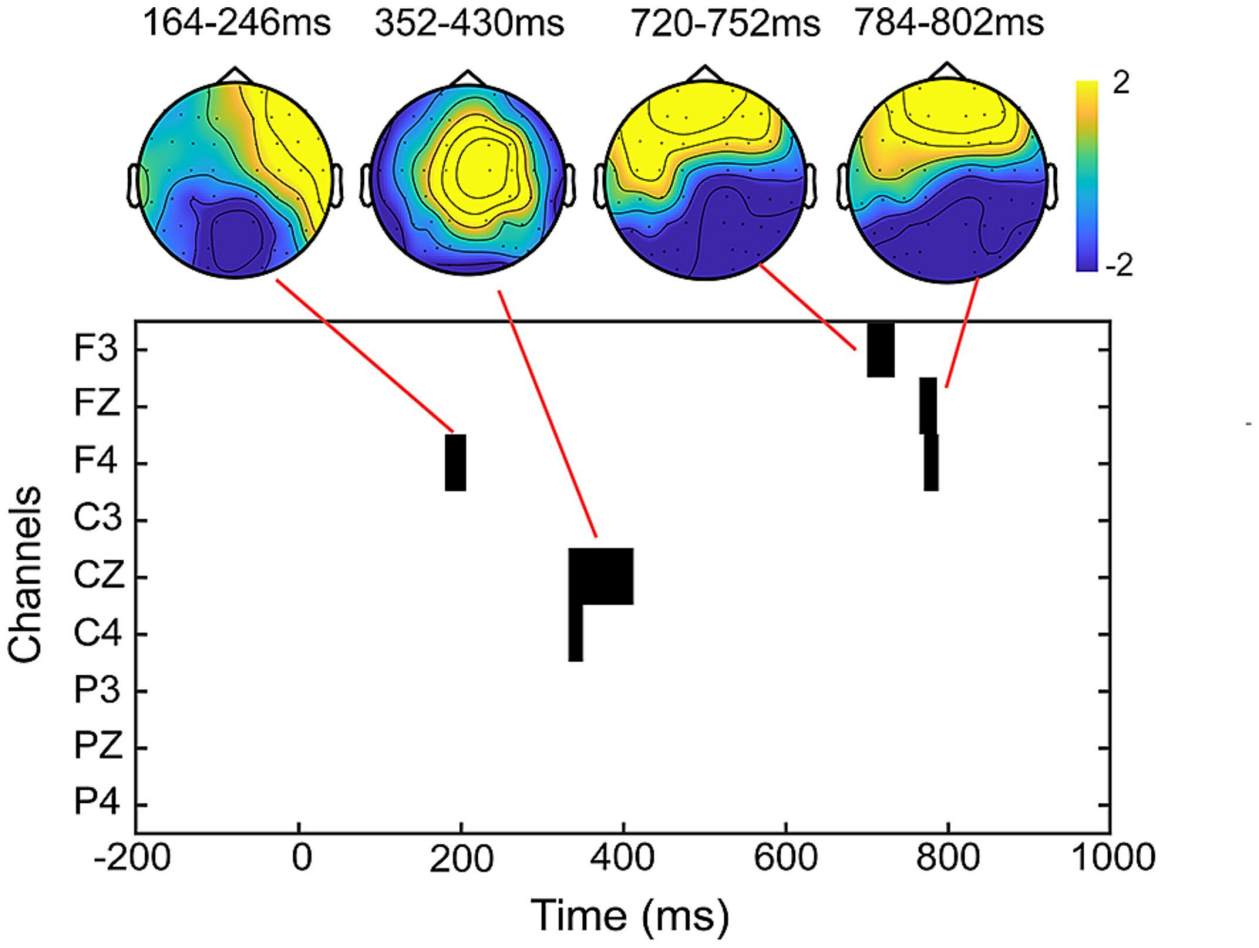
Results of the cluster-based permutation test. The clusters and corresponding topographical maps revealed significant differences in ERP responses between peer-feedback and non-feedback context in the time windows of 164–246 ms, 352–430 ms, 720–752 ms, and 784–802 ms.

The results of the cluster-based permutation test revealed significant differences between peer-feedback and non-feedback context in the following time window: 164–246 ms (*p* = 0.020), 352–430 ms (*p* < 0.001), 720–752 ms (*p* = 0.009) and 784–802 ms (*p* = 0.027). Based on the ERP topographical maps, the ERP responses in the time windows of 720–752 ms and 784–802 ms indicated the same ERP component. These components align with our pre-specified ERP components identified through previous studies and visual inspection (see Figure 5).

### 3.3 Functional brain network analysis

#### 3.3.1 General functional brain network characteristics

As shown in Table 3, the analysis of the general functional brain network characteristics revealed significant differences in the AC of the brain network between the peer-feedback and non-feedback contexts in both the delta and beta bands. Specifically, the brain network displayed a higher AC under the peer-feedback context compared to that under the non-feedback context in the delta band. Similarly, in the beta band, participants exhibited higher AC and lower LE of the brain network when exposed to peer-feedback than non-feedback. No significant effects were observed in the other frequency bands analyzed.

**Table 3:** Mean (SD) and *t*-test results of brain network characteristics.

	Brain network characteristics	*df*	Peer-feedback	Non-feedback	*t*	*p*	*Cohen’s d*
Delta	CC	26	0.28 (0.02)	0.28 (0.02)	0.24	0.814	0.09
	AC	26	**0.19 (0.05)**	**0.17 (0.05)**	**2.65**	**0.017**	**1.04**
	GE	26	0.24 (0.02)	0.24 (0.02)	−1.65	0.110	0.65
	LE	26	0.34 (0.01)	0.34 (0.01)	−1.31	0.202	0.51
Theta	CC	26	0.28 (0.02)	0.28 (0.03)	<0.001	1	<0.001
	AC	26	0.19 (0.05)	0.17 (0.04)	−0.86	0.395	0.34
	GE	26	0.24 (0.01)	0.24 (0.01)	0.33	0.746	0.13
	LE	26	0.34 (0.01)	0.35 (0.01)	−1.14	0.265	0.45
Alpha	CC	26	0.28 (0.03)	0.28 (0.03)	−0.36	0.726	0.14
	AC	26	0.18 (0.05)	0.18 (0.04)	0.06	0.955	0.02
	GE	26	0.24 (0.01)	0.24 (0.01)	−0.10	0.923	0.04
	LE	26	0.35 (0.01)	0.35 (0.01)	−0.39	0.703	0.15
Beta	CC	26	0.28 (0.02)	0.28 (0.03)	−1.51	0.144	0.59
	AC	26	**0.19 (0.05)**	**0.17 (0.04)**	**2.64**	**0.014**	**1.04**
	GE	26	0.24 (0.02)	0.24 (0.01)	0.63	0.534	0.25
	LE	26	**0.34 (0.01)**	**0.35 (0.01)**	**−2.59**	**0.016**	**1.02**

CC, clustering coefficient; AC, assortativity coefficient; GE, global efficiency; LE, local efficiency. Significant effects are marked in bold.

**Figure 6:**
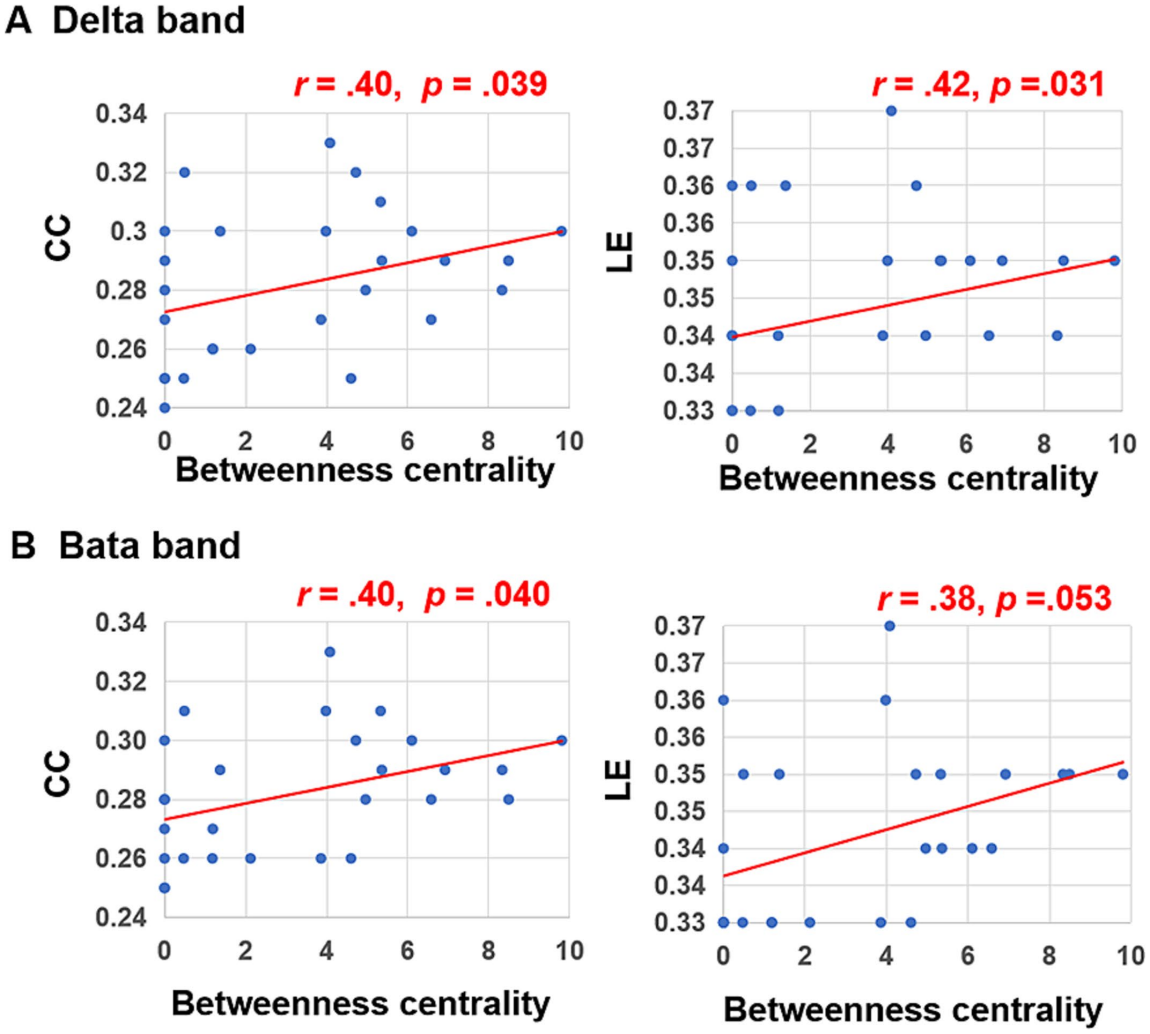
Correlations between betweenness centrality and clustering coefficient (CC), local efficiency (LE) of functional brain networks in delta **(A)** and beta bands **(B)**.

Subsequent Person correlation analyses were conducted to explore the associations between the graph characteristics of brain networks in delta and beta bands, which showed significant differences between the peer-feedback and non-feedback contexts, and individual social network metrics. The results indicated significant or marginally significant positive correlations between CC (delta band: *r =* 0.40, *p =* 0.039; beta band: *r =* 0.40, *p =* 0.040) and LE (delta band: *r =* 0.42, *p =* 0.031; beta band: *r =* 0.38, *p =* 0.053) of brain networks under the peer-feedback context with betweenness centrality. Notably, no statistically significant relationships were observed under the non-feedback context (see Figure 6). However, no correlations remained significant after FDR correction.

#### 3.3.2 Functional connectivity analysis

**Figure 7:**
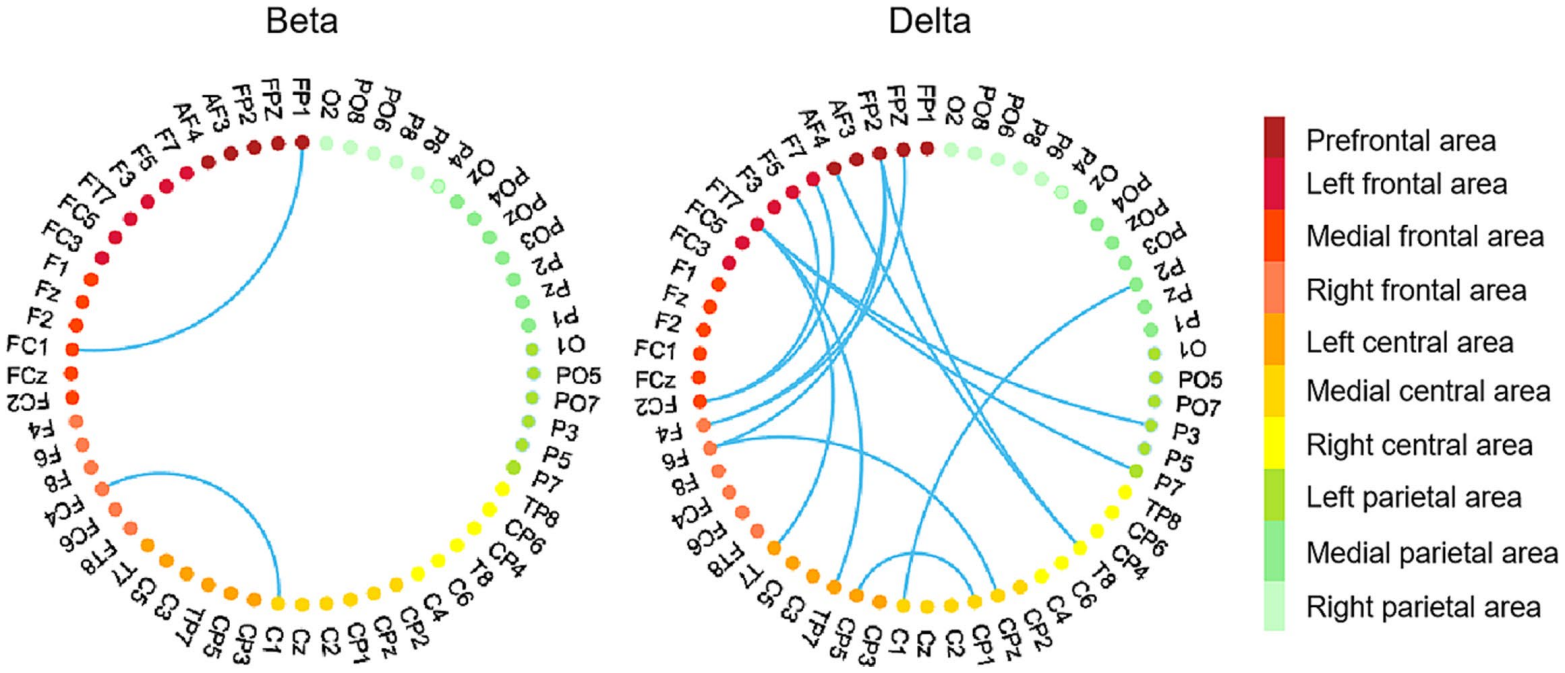
EEG channel pairs exhibiting significant correlations between ΔPLV values (peer-feedback minus non-feedback) and betweenness centrality. In each subfigure, the nodes with different colors represent EEG channels located in different areas. Red nodes, frontal area; orange nodes, central area; green nodes, parietal area. Network-based statistics uncorrected *p* < 0.01.

Given that only betweenness centrality displayed significant correlations with brain network graphic metrics in the beta and delta bands, we focused our further analysis on betweenness centrality. Specifically, we conducted Pearson correlation analyses between ΔPLV and betweenness centrality in the beta and delta bands, respectively. Individuals with high betweenness centrality exhibited enhanced ΔPLV values between pairs of EEG channels located within the frontal area and across brain areas (such as frontal and parietal area, frontal and central area), especially in the delta band (see Figure 7).

## 4 Discussion

In the present study, we used EEG technology to investigate whether and how the effects of group norms on decision-making are modulated by an individual’s social network structure. Well-connected individuals with large real-life social network size or high betweenness centrality exhibited increased amplitudes on N2_diff_, P3_diff_, and LPP_diff_ when learning that others’ opinions differed from theirs, indicating heightened sensitivity to self-group conflicts. Increased efficiency in functional brain networks in the delta and beta band under the social conflict was observed in individuals with high betweenness centrality. Moreover, individuals with high betweenness centrality exhibited higher synchronization between the frontal and parietal lobes. These results indicated that an individual’s role in the social network impacts sensitivity and processing efficiency to group norms for exercise behaviors.

### 4.1 ERPs effects: N2, P3, and LPP

Our results revealed N2, P3, and LPP effects for the peer-feedback context compared to the non-feedback context. More importantly, the amplitudes of N2_diff_, P3_diff_, and LPP_diff_ were modulated by an individual’s social network structure, such as real-life social network size, out-degree centrality, and betweenness centrality.

Our study has revealed that the differences in N2 amplitude between conditions involving peer-feedback and non-feedback are primarily localized to the parietal lobe (Aral and Nicolaides, 2017; Wang et al., 2019). This posterior-contralateral component (N2pc) is commonly associated with allocating additional attention resources to automatically manage or monitor conflicts (Liu et al., 2021). In the present study, encountering group norms that contradict an individual’s expectations can engender a kind of social conflict, given that violating these norms may result in punishment such as social exclusion (Klucharev et al., 2009). Previous studies have predominantly linked the FRN component, which typically peaks within the anterior cingulate cortex (ACC), to monitoring others’ feedback information in social domains and detecting social conflicts (Baker and Holroyd, 2011; Glazer et al., 2018; Qiu et al., 2019). Contrary to existing literature focused on FRN, our finding revealed a clear negative deflection in the posterior parietal lobe, i.e., N2pc. We believe that this N2pc activation is an early neural response preceding the FRN and is associated with the mobilization of attention resources caused by social conflicts instead of social conflicts and unexpected outcomes detection. Previous studies have provided evidence of a more negative-going N2pc when individuals are confronted with violations of group norms, unexpected stimuli, or perceptual mismatch (Folstein and Petten, 2008; Wang Z. et al., 2020; Xie et al., 2016; Liu et al., 2021). We found the N2_diff_ amplitude negatively correlated with individuals’ social network metrics, indicating that well-connected individuals may allocate more attention resources toward socially conflicting information.

A centro-parietal P3 following a feedback stimulus is suggested to reflect the attention-driven integration of various aspects of the current situation, such as outcome, affective information, and motivation, into working memory to make decisions (Glazer et al., 2018). We found a positive correlation between real-life social network size and P3 amplitude. Real-life social network size can better reflect the quality of an individual’s social relationship, which is usually considered an indicator similar to personality traits (Baek et al., 2020; Smith and Christakis, 2008).

Many studies based on the Social Brain Hypothesis have found significant correlations between individual social network size and brain activities underlying cognitive tasks (Brashears et al., 2020; Stiller and Dunbar, 2007). Higher amplitudes of N2_diff_, P3_diff_, and LPP_diff_ for individuals with large social network sizes may be due to higher levels of individual social function or social cognitive capabilities processing social conflicts. In-class social network metrics are calculated using graph theory approaches and describe an individual’s structural position within a social group, such as whether they occupy a core or peripheral role. However, these metrics may only capture a part of an individual’s social life. The lack of significant correlation between in-class social network metrics and P3_diff_ amplitude may suggest that an individual’s position within a social group may not directly influence cognitive processes associated with decision-making. Other variables such as social status or identification within the group may confound this relationship. Although there are positive correlations among real-life social network size and in-class social network metrics, they may represent different aspects of an individual’s social life. Future research could differentiate the significance of these different social network metrics.

In addition, the peer-feedback context evoked a more positive-going LPP wave than the non-feedback context in the frontal lobe. The LPP_diff_ amplitude in the frontal lobe further revealed a positive relationship with an individual’s social network metrics. The LPP is the final ERP component during the processing of group norms, which reflected the encoding of social feedback-related emotions with a more positive LPP following negative feedback than positive feedback (Glazer et al., 2018; Kivity and Huppert, 2019). It is suggesting that individuals at the centre of the network, such as those with more real-life friends and higher out-degree and betweenness centrality within the group, may experience more emotional reactivity and regulation when they violate group norms.

Our research has revealed a connection between social network metrics and the dynamic processing of group norms. We have found that well-connected individuals exhibit a heightened sensitivity toward group norms by paying more attention, exerting more effort in cognitive processing, and experiencing heightened emotional responses when confronted with social conflicts. It indicates that identifying and processing group norms is essential for individuals to maintain their social relationships and status. Well-connected individuals may perceive their core position as threatened when faced with inconsistent opinions from others. Therefore, they may consolidate their positions by carefully considering the opinions of others. Interestingly, we have revealed that betweenness centrality was negatively associated with conforming behaviors, suggesting that individuals may evaluate the pros and cons of their choices about other information, such as their status, identity, and social motives. These findings can provide valuable insights into the complex nature of social interaction and help understand how individuals navigate the challenges of group dynamics.

### 4.2 Social network and functional brain network

Our recent study revealed that increased CC and LE of functional brain networks constructed based on the phase synchronization of brain activity in the delta and beta bands are associated with an individual’s betweenness centrality. This association suggests that individuals with higher betweenness centrality might possess more efficient cognitive processing regarding group norms.

Previous research has established a significant correlation between brain and social networks (Bickart et al., 2011, 2012, 2014; Kanai et al., 2012). However, these studies primarily focused on specific brain structures and their functions. Our current work explores brain function based on graph theory analysis, offering a comprehensive perspective on the brain’s working patterns during cognitive tasks. The topological properties of complex networks, mainly CC and LE, enable a detailed description of the brain’s functional segregation. This segregation reflects the ability to undertake cognitive processes within specialized brain regions efficiently. Prior research has associated enhanced functional segregation with simple cognitive tasks (Cohen and D’Esposito, 2016; Braun et al., 2015; Wang et al., 2019; Shine et al., 2016). Thus, in the present study, the increased functional segregation of brain networks for well-connected individuals may indicate higher efficiency and density of specialized brain regions.

We found increased functional segregation of brain networks for well-connected individuals, mainly in the delta and theta bands. Delta and beta power following feedback are sensitive to performance and reward evaluation (Cavanagh, 2015). Several studies suggest that, in contrast to negative feedback, such as loss or unexpected stimuli, positive feedback could invoke a higher power of delta and a lower power of beta, which are associated with subsequent behavioral adjustments to improve performance (Bernat et al., 2015; Cavanagh, 2015; Luft, 2014). While earlier studies have primarily measured delta and beta power in the immediate aftermath (approximately 100–600 ms) of feedback presentation, our research extends these findings by highlighting the role of functional connectivity in the theta and beta bands in processing group norms more comprehensively.

Our study emphasizes the critical role of functional brain networks, especially in the delta and beta bands, in understanding how individuals process group norms. While we examined multiple social network metrics, only betweenness centrality showed a significant link to the characteristics of functional brain networks. Betweenness centrality indicates an individual’s potential to disseminate information and influence others. Individuals with high betweenness centrality often confront more complex social scenarios, such as balancing the interests and opinions of diverse groups or individuals in their daily lives. Social cognitive skills are well-trained in these individuals. Our findings suggest an individual’s betweenness centrality may linked more to group norm processing efficiency than other social network metrics. However, more empirical studies are needed in the future to confirm this.

### 4.3 Social network and functional connectivity

Individuals with high betweenness centrality showed increased functional connectivity both within the frontal area and across different brain areas, especially between the frontal and parietal areas as well as between the frontal and central areas, particularly in the delta band. It implies that well-connected individuals might be more capable of allocating cognitive resources across different brain regions. Previous research has emphasized the significant role of the frontal area in social functioning. Notably, an individual’s social network size is linked to the strength of functional connectivity among the dorsal medial prefrontal cortex, the dorsolateral prefrontal cortex, and the orbitofrontal cortex in the resting state (Noonan et al., 2018; Zhang et al., 2021; Zhang et al., 2022). Furthermore, the capacity to perceive and interpret social signals has been found to strongly correlate with the level of functional connectivity between the frontal and parietal lobes (Greene et al., 2009; Moratti et al., 2004). Our research indicated that synchronization within frontal area and across different brain areas may be vital for an individual’s ability to maintain social connections.

Our study demonstrated that the impacts of group norms on individuals’ attitudes and behaviors can be modulated by an individual’s position within the group. Specifically, individuals with a central position exhibited heightened sensitivity to group norms. These findings underscore the importance of considering individuals’ social connections within the group if utilizing group norms to promote healthy behaviors, such as exercise. Additionally, the capability to recognize and understand group norms appears to play a crucial role in distinguishing well-connected individuals from others. It indicates that enhancing understanding of group norms and their importance may improve the social network structure of those less-connected individuals.

### 4.4 Limitation

The present study further clarified the relationship between social network structure and neural activities underlying dynamic group norm processing. Our findings support those well-connected individuals exhibited enhanced sensitivity and efficiency in processing group norms. These findings offer a profound insight into the causes behind variations in individual social network structures. They are valuable for developing intervention strategies to enhance people’s social world. However, this study has several limitations. Firstly, in the present study, after FDR correction (*p* < 0.05), no correlation results retained their statistical significance. Even though the *p*-values after FDR correction fall short of expectations, as an exploratory study, we highlight the discernible tendency in how brain activities during group norm processing are influenced by the individuals’ social network structure. More research is needed in the future to further investigate and verify this hypothesis. Secondly, in this study, we used a socio-centric network to depict individuals’ social relationships, which required us to gather social relations and EEG indicators of all individuals in the group. However, it was a challenge for us to ensure that most of the group participated in the EEG experiment, which led to a constrained sample size in our study. Expanding our participant pool to include more groups might introduce group variables such as group cohesion, potentially affecting our findings. Future studies with a larger sample size are needed to examine our findings. Thirdly, the present study was conducted on only one group selected at random. Future studies should consider recruiting more groups and incorporating group characteristics into the analysis, for example, examining how the density of social relationships within a group affects the individual processing of social information. Fourthly, our findings further suggest that group norms can influence individuals’ willingness to exercise. Nonetheless, the effectiveness of social influence in promoting exercise willingness into behaviors needs further exploration. Exercise behaviors might stem from personal preferences or thoughtful consideration, suggesting that the influence of group norms might be limited. To enhance the validity of our findings in a broader social norm perception, future studies could explore additional social conformity domains, including dietary habits, consumer behavior, emotion perception, etc. Fifth, we did not focus on the behavioral differences between subgroups and the whole group. Such differences could lead to group behavior exerting distinct social influences on members of different subgroups. Future studies should take this difference into account when investigating group norms. Finally, while the principal findings of this research are derived from correlational data, introducing studies to establish causality could enhance understanding of the relationship between social networks and social information processing.

## 5 Conclusion

This study is one of the first to directly examine the neural basis of how an individual’s network structure interacts with the dynamic processing of group norms. It reveals that people adjust their exercise decisions based on the group’s choice. Furthermore, well-connected individuals in the group display enhanced sensitivity and efficiency in brain network connectivity across various neurocognitive processes when processing social information. These findings have implications for further studies concerning the evolution of social network structures and their impact on individual behaviors.

## Data Availability

The raw data supporting the conclusions of this article will be made available by the authors, without undue reservation.

## Footnotes

^1^ http://www.nitrc.org/projects/gretna/
