# Supplementary material for: Social network structure modulates neural activities underlying group norm processing: evidence from event-related potentials
Source: Front Hum Neurosci. 2024 Nov 13;18:1479899. doi: 10.3389/fnhum.2024.1479899 (PMC11599178; doi:10.3389/fnhum.2024.1479899)
Supplement: Supplementary file 1 [file Data_Sheet_1.docx]

Supplementary Material

**1 The Development of Experimental Stimuli**

A comprehensive set of 60 color images depicting distinct exercise behaviors were used as the experimental stimuli. Before the formal experiment, a preliminary evaluation was conducted involving 30 participants (10 males; mean age = 21.74 years, *SD =* 2.66 years), who neither took part in the initial social network questionnaire survey nor the subsequent EEG experiment. These participants were asked to evaluate each stimulus’s arousal, valence, and exercise intensity levels separately using a 7-point Likert scale ranging from 1 (indicating very calm, negative, easy) to 7 (indicating very excited, positive, strenuous).

All the stimulus images displayed moderate ratings across the three evaluated dimensions: arousal (*M* ± *SD*: 4.33 ± .43), pleasure (*M* ± *SD*: 4.58 ± .74), and exercise intensity (*M* ± *SD*: 4.62 ± .90). The stimulus images were then randomly assigned to two conditions: peer-feedback condition and non-feedback condition. Thirty stimulus images for each condition. The images’ arousal, valence, and exercise intensity levels in both conditions were balanced and matched (see Table S1).

**Table 1 The ratings of arousal, pleasure, and exercise intensity on stimulus pictures in peer-feedback and non-feedback condition**

|  | **Arousal** | **Valence** | **Exercise intensity** |
| --- | --- | --- | --- |
| Peer-feedback condition | 4.41 ± .43 | 4.58 ± .76 | 4.63 ± .98 |
| Non-feedback condition | 4.25 ± .42 | 4.59 ± .73 | 4.62 ± .82 |
| *t* | 1.44 | -.03 | .08 |
| *p* | .157 | .973 | .938 |

**2** **Behavior adjustment between initial rating and final rating**

We performed a two-way repeated-measures ANOVA with the factors of different ratings (initial rating and final rating) and social influence direction (positive feedback: peer ratings higher than participant ratings; negative feedback: peer ratings lower than participant ratings; non-feedback: no peer ratings presented).

The main effects of ratings (*F*(1, 26) = 5.18, *p* = 0.031, η²_p_ = 0.17), social influence direction (*F*(2, 52) = 35.20, *p* < 0.001, η²p = 0.58), and their interaction (*F*(2, 52) = 11.40, *p* = 0.002, η²_p_ = 0.31) were all significant. Subsequent post-hoc analysis revealed a significant effect of negative feedback (*F*(1, 26) = 23.74, *p* < 0.001, η²_p_ = 0.48; mean ± SE: initial rating, 6.18 ± 0.14; final rating, 5.84 ± 0.17) and a significant effect of non-feedback (*F*(1, 26) = 6.08, *p* = 0.021, η²_p_ = 0.19; mean ± SE: initial rating, 4.98 ± 0.17; final rating, 5.12 ± 0.17).

These results suggest that peer feedback has a significant impact on individual decision-making, especially in the context of negative feedback. Future studies should consider the direction of social influence in conjunction with brain responses and measures of social network centrality.
